# Influence of Different Light-Emitting Diode Colors on Growth and Phycobiliprotein Generation of *Arthrospira platensis*

**DOI:** 10.3390/life12060895

**Published:** 2022-06-15

**Authors:** Conrad H. G. Jung, Peter Waldeck, Shadi Sykora, Steffen Braune, Ingolf Petrick, Jan-Heiner Küpper, Friedrich Jung

**Affiliations:** 1 Carbon Biotech Social Enterprise AG, 01968 Senftenberg, Germany; c.jung@carbonbiotech.eu (C.H.G.J.); jan-heiner.kuepper@b-tu.de (J.-H.K.); 2 Institute of Materials Chemistry, Thermodynamics, Brandenburg University of Technology Cottbus-Senftenberg, 01968 Senftenberg, Germany; peter.waldeck@b-tu.de (P.W.); ingolf.petrick@b-tu.de (I.P.); 3 Experimental Physics, Mechanical Engineering, Electrical and Energy Systems, Brandenburg University of Technology, 01968 Senftenberg, Germany; shadi.sykora@b-tu.de; 4 Institute of Biotechnology, Molecular Cell Biology, Brandenburg University of Technology Cottbus-Senftenberg, 01968 Senftenberg, Germany; steffen.braune@b-tu.de; 5 Faculty of Health Sciences Brandenburg, Brandenburg University of Technology Cottbus-Senftenberg, 01968 Senftenberg, Germany

**Keywords:** *Arthrospira platensis*, Spirulina, colored illumination, light emitting diode, LED, phycobiliprotein, biomass, yield

## Abstract

Light-emitting diodes (LED) can be utilized as tailorable artificial light sources for the cultivation of cyanobacteria such as *Arthrospira platensis* (AP). To study the influence of different LED light colors on phototrophic growth and biomass composition, AP was cultured in closed bioreactors and exposed to red, green, blue, or white LED lights. The illumination with red LED light resulted in the highest cell growth and highest cell densities compared to all other light sources (order of cell densities: red > white > green > blue LED light). In contrast, the highest phycocyanin concentrations were found when AP was cultured under blue LED light (e.g., order of concentrations: blue > white > red > green LED light). LED-blue light stimulated the accumulation of nitrogen compounds in the form of phycobiliproteins at the expense of cell growth. The results of the study revealed that exposure to different LED light colors can improve the quality and quantity of the biomass gained in AP cultures.

## 1. Introduction

The increase in the world’s population in combination with a climate change-induced decrease in fertile arable land has been counterbalanced by the intensification of agriculture and maritime economy over recent decades [1,2]. Now, the protein supply is gradually being jeopardized, particularly in developing countries [3]. One example is the drastic collapse of cod stocks in Newfoundland (since 1992), which has not recovered, despite an absolute ban on fishing and farming activities [4]. The situation is similar for cod stocks in the North Sea and the Baltic Sea. Furthermore, the rice production is anticipated to decline by up to 40% by the end of this century [5]. Losses of food stock are expected in other regions as a result of the desertification of arable land [2,6].

Thus, one of the greatest global challenges is the supplementation of the world’s population with protein-rich food [7]. To ensure protein supply, *Arthrospira platensis* (AP, commonly referred to as *Spirulina platensis* or Spirulina microalgae) are particularly suitable as a renewable raw material, as they are extremely superior to plant protein production [8,9]. Total yields estimated for the Lake Texcoco in Mexico or production plants in Israel range between 40 tons/ha per year (*A. maxima*) and 60–70 tons/ha per year (*A. platensis*), respectively [8,9,10].

AP produces a significantly higher yield of protein per hectare than conventional agricultural crop production methods (Protein yield *A. maxima*: 28 tons/ha per year, *A. platensis*: 39–45 tons/ha per year) [8,9,10]. About 250 times more protein can be produced on one hectare compared to the intensive rearing of cattle [11]. Moreover, the cultivation of AP is possible on land that cannot be used for agricultural purposes. The production of 1 kg of protein by AP requires 7.4 times less water compared to soy and 14.4 times less water is needed for cultivation compared to intensive cattle farming [12,13,14].

AP has a very high protein content of 60–70% of dry matter (about 30–35% for soy). The amino acid composition of the proteins obtained from AP is nutritionally close to the World Health Organization (WHO)/Food and Agriculture Organization of the United Nations (FAO) standard for an optimal protein composition [15]. Thus, AP is considered one of the most nutritious foods in the world [16], with proteins containing all essential amino acids that meet the requirements of the Food and Agriculture Organization (FAO) [17]. AP is also rich in B-vitamins, iron, magnesium, potassium and many other vitamins and minerals, as well as antioxidants. The cyanobacterium is generally considered safe as a food by the European Food Safety Authority (EFSA) [15]. In addition, AP contains an abundant source of different classes of ingredients [18] of interest for nutraceutical applications [19,20,21] or even as pharmaceuticals with potential biological effects on tissue or blood cells [22,23,24,25]. Due to the very alkaline pH, AP cultures can be maintained without the supplementation of biocide treatments in the growth medium, which is a clear advantage with respect to food safety and environmental issues.

Worldwide, AP is produced in open or covered ponds, with sunlight as the primary photosynthetic energy source. Although sunlight is more cost-effective, illumination with artificial light sources, such as light-emitting diodes (LED), is still economically feasible when the generated biomass is used as a feedstock for high-value products, such as phycocyanin, carotenoids, n-3-polyunsaturated fatty acids or nutraceuticals [26,27].

Studies on optimizing the production yield of AP show that, among other factors, the light color (and of course intensity [28]) can influence the build-up of biomass but also of various ingredients [29,30,31]. On a molecular level, pigments and phycobiliproteins play a pivotal role for these processes. In cyanobacteria, the green gap in the absorption spectrum of chlorophyll-a is filled by phycobiliproteins, which show a high absorption in a broad wavelength range (495–650 nm) [32]. Phycoerythrin (PE) absorbs green wavelengths (495–570 nm), while phycocyanin (PC) and allophycocyanin (APC) absorb green-yellow (550–630 nm) and orange-red (650–670 nm) wavelengths, respectively [33].

Thus, it appears feasible that the biomass productivity of AP can be significantly influenced by the light spectrum of the light source applied for the cultivation. However, the literature data regarding this aspect are not entirely clear [30,34,35,36,37,38,39]. Therefore, the influence of LED light color on the biomass and various ingredients of AP was studied within the scope of this work.

## 2. Materials and Methods

### 2.1. Microalgae Strain and Growth Medium

The *Arthrospira platensis* strain (SAG 21.99) used for cultivation experiments was obtained from the Department of Experimental Phycology and Culture Collection of Algae at Goettingen University (EPSAG). The stock cell suspension of AP was cultured in fresh water supplemented with Zarrouk medium [40]. Zarrouk medium consisted of the following ingredients (per liter): 16.8 g NaHCO_3_, 0.5 g K_2_HPO_4_, 2.5 g NaNO_3_, 1 g K_2_SO_4_, 1 g NaCl, 0.2 g MgSO_4_·7H_2_O, 0.04 g CaCl_2_, 0.01 g FeSO_4_·7H_2_O, 0.08 g Na_2_EDTA and 1 mL of trace metal solution. The trace metal solution consisted of (per liter): 2.86 g H_3_BO_3_, 1.81 g MnCl_4_·4H_2_O, 0.22 g ZnSO_4_·4H_2_O, 0.0177 g Na_2_MoO_4_, 0.079 g CuSO_4_·5H_2_O. The growth medium was initially sterilized at 121 °C in an HV-50 autoclave (SYSTEC VX-95, Systec GmbH, Linden, Germany) for 15 min.

### 2.2. Experimental Design

**Figure 1:**
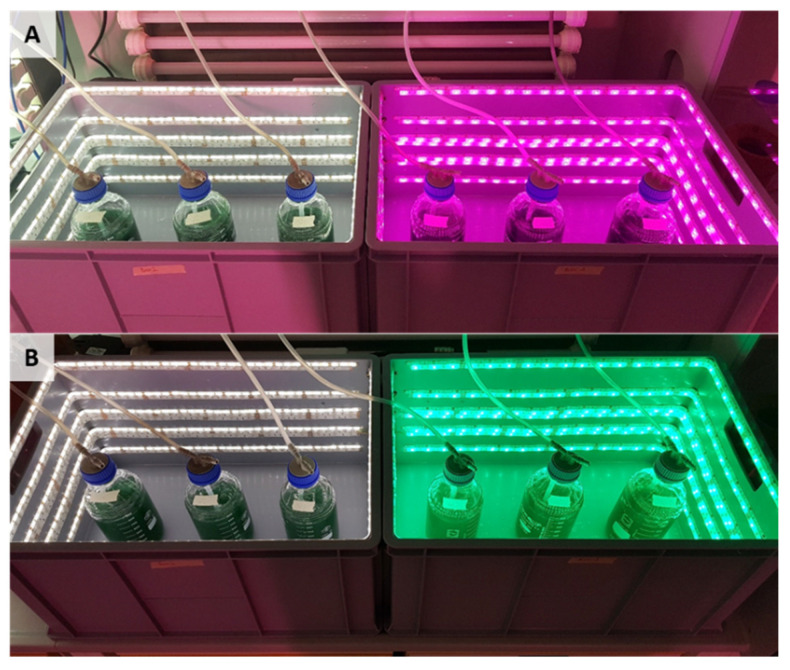
Representative images of light boxes with white, (**A**) red and (**B**) green LED lights. Each experiment comprised three glass bioreactors of *Arthrospira platensis*, strain SAG 21.99 per LED type.

**Figure 2:**
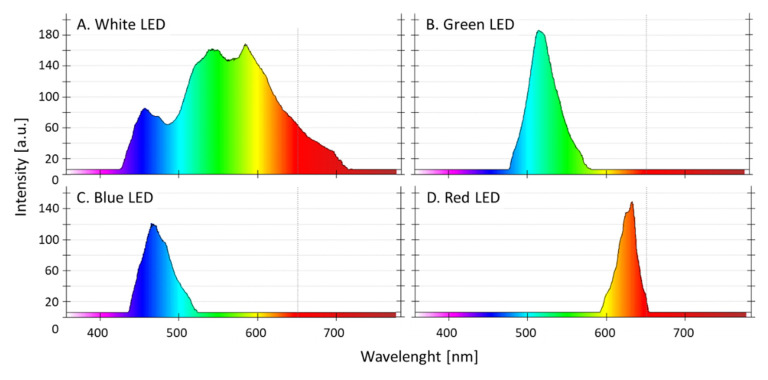
Light spectra of the four LED lamps: (**A**) white LED light, (**B**) green LED light, (**C**) blue LED light and (**D**) red LED light (5050 SMD LEDs, spectra were determined with a spectrometer Kvant, 21-2301 Spectra 1, Spectral range: 360–940 nm; Bratislava, Slovakia).

For all experiments, AP was taken from a backup bubble column bioreactor, which was in the stationary phase. In this backup reactor, the cells were cultured in Zarrouk full medium with an aeration rate of 20 L/h. The series of experiments (*n* = 3 in parallel) were conducted in aerated (20 L/h) glass bioreactors with Zarrouk full medium (Figure 1). The photon flux density was 12 µE/(m^2^∙s). The temperature in the bioreactors was 25 °C. The cultivation time in the full Zarrouk medium was 8.7 days. Stirring was carried out by ambient air injection, which was pumped through a membrane filter (Millipore; 0.45 µm pore size, 10 cm diameter) and with an air bubble diameter of 1.5 cm. As light sources, white, red, green, and blue LEDs (5050 SMD LEDs) were used. Figure 1A,B show the light boxes in which the bioreactors were illuminated. The peak wavelength emissions (λ) from the LED light colors used in the study were at 466.9 nm for the blue LED light, at 514.1 nm for the green LED light and at 632.9 nm for the red LED light. The white LED light exhibited multiple peaks in the blue and green wavelength range (see Figure 2). The light intensity could be varied by pulse-width modulation via a microcontroller. The same photon flux density was adjusted and lowered to the maximum capability of the lowest one (green), so that the light intensity was equal for all cultures (on average, 12 µE/(m^2^∙s) near the bioreactor surface). The glass bioreactors were illuminated 24 h per day.

The emission spectra of the four LEDs were measured using a spectrometer (Kvant, 21-2301 Spectra 1, Spectral range: 360–940 nm; Bratislava, Slovakia; Figure 2).

The light intensity was measured using an LI-250 light meter with an LI-190SA pyranometer sensor (LI-COR, Inc., Lincoln, NE, USA). The optical density (OD, Thermofisher, Genesys 100 Bio, Waltham, MA, USA), temperature (PT1000, Wernberg, Germany), and pH values of the culture medium (EGA 133, Sensortechnik Meinsberg, Meinsberg, Germany) were measured every day during the cultivation time.

### 2.3. Determination of Arthrospira Platensis Dry Weight

Cell pellets were prepared by centrifugation at 17,000× *g*. The pellets were washed with distilled water and centrifuged a second time. The pellets remained in the glass tubes. After removing the aqueous supernatant, they were dried in an oven at 103 °C for 24 h. Finally, the dried biomass was weighed.

### 2.4. Preparation of Arthrospira Platensis Extracts for Pigment Measurements

AP dry biomass (100 mg AP powder/25 mL extract volume) was stirred once for 48 h at 4 °C in sterile phosphate buffered saline (onefold, B. Braun, Melsungen, Germany). Afterwards, the extract was centrifuged at 17,000× *g* for 20 min. The extract was stored at −80 °C until further processing.

The concentrations of PC, PE and the APC (mg/mL) were calculated according to the following equations [41]:

PC [mg/mL] = (OD_615_−0.474∙OD_652_)/5.34

APC [mg/mL] = (OD_652_−0.208∙OD_615_)/5.09

PE [mg/mL] = (OD_562_−2.41∙PC−0.849·APC)/9.62

OD_562_, OD_615_ and OD_652_ represent the optical absorptions at the wavelengths of 562 nm, 615 nm and 652 nm, respectively.

### 2.5. Statistical Analysis

For all samples, the arithmetic means ± standard deviations are given. The yield under the different colored LEDs was related to the yield under white light. The differences were tested against white light using paired *t*-tests. Significance levels lower than 0.05 were considered significant.

## 3. Results

**Figure 3:**
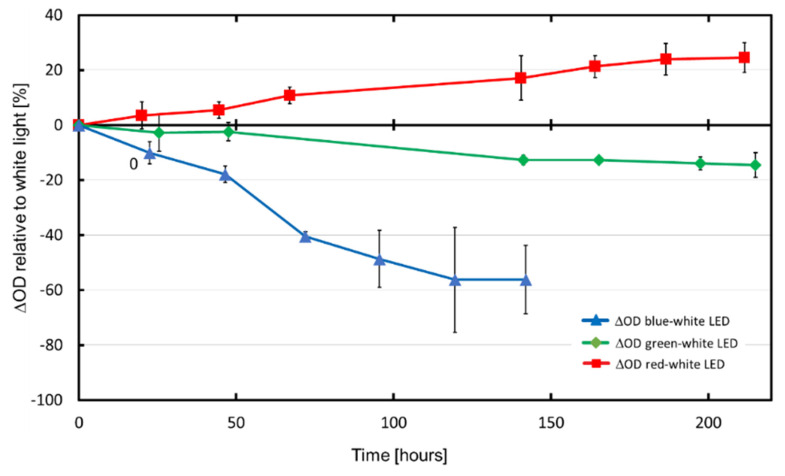
Normalized mean differences in optical densities of *Arthrospira platensis* cultures during the cultivation period of 8.7 days.

As experiments were conducted for a period of three months, the experimental conditions such as the room temperature, scattered light intensity (from other neighboring experiments) and CO_2_ concentration in the room air varied slightly over time, which could influence the growth of AP. These variations were compensated by calculating the differences in the optical densities at 760 nm (ΔOD) of AP produced under colored LED light and those produced under white LED light (measured in parallel in each individual experiment). These values were normalized to the ODs of the respective white light (Normalized ΔOD = (OD_color_ − OD_white_)/OD_white_·100). In each case, three bioreactors illuminated with colored light and three bioreactors illuminated with white LED light were averaged. Figure 3 shows the biomass yield after the exposure of AP to different LED-light colors in relation to the yield after white LED light illumination (normalized ΔOD values).

At the end of the cultivation period, more biomass (+24.50%, *p* = 0.0052) was achieved with red LED light illumination in comparison to white LED light. On the other hand, the exposure of AP to blue LED light resulted in a significantly lower biomass yield (−56.28%, *p* = 0.00134; the cultivation was stopped after 141 h) than during illumination with white LED light. Additionally, when illuminated with green LED light, significantly less AP biomass (−14.56%, *p* = 0.00134) was gained compared to the illumination with white LED light.

The dry weight biomasses collected at the end of the cultivation period of 8.7 days are compiled in Table 1A. The coefficients of variation (CV) for the dry weight biomasses ranged from 0.038 to 0.080 between the four experiments with the white LED light. The highest CVs were observed after the illumination of the cultures with blue LED light (CV = 0.216). The illumination with green LED light (CV = 0.139) and red LED light (CV = 0.102) revealed lower CV values compared to blue LED light but were higher compared to the values obtained with white LED light.

**Figure 4:**
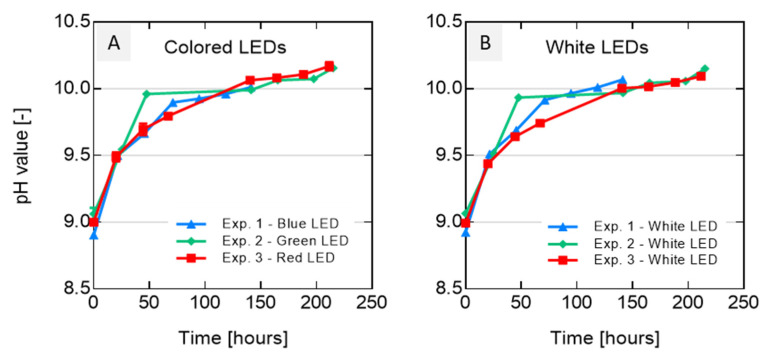
The pH values of the three experiments for (**A**) the red, blue, and green LEDs as well as (**B**) the white LED lights (cultivation period of 8.7 days, *n* = 12). Each experiment is displayed by different symbols, means and standard deviations. The respectively colored interconnecting lines represent linear trendlines between single datapoints for each LED light color.

The pH values over cultivation time (as average values over the three experiments in each case) are shown in Figure 4A for colored LEDs and Figure 4B for white LEDs for each of the three experiments. For all bioreactors, the pH values remained about pH 9 and reached a pH of 10 after 60 h. The values remained at this level until the end of the cultivation period. The pH value increase during the illumination with white LED light was very similar to what was observed for the colored LEDs. Thus, differences between the single experiments can hardly be distinguished in the diagram (Figure 4).

This confirms that the LED light color only had minor influences on the pH values. To understand the relations between pigment concentrations under light sources with different wavelengths, the PC, APC and PE contents were measured.

Table 1B shows the pigment concentration in percent related to the illumination with white LED light. The illumination with blue LED light led to a marked increase of phycocyanin—in relation to warm white light, as a model for sunlight—(+15.73%, *p* < 0.001), while it decreased under green LED (−8.53%, *p* < 0.001) or red LED lights (−6.97%, *p* < 0.001).

The generation of APC was not affected differently under blue-, green-, and red-LED light illumination in comparison to white LED light. The PE concentration only increased during the illumination with blue LED light (14.72%, *p* < 0.001, differently to white light) but remained unchanged during the green or red LED light illumination.

## 4. Discussion

The study provides important insights for the cultivation of AP under illumination with artificial LED light sources. In Europe, large amounts of microalgae are required to refill production ponds, especially in spring, as this requires about 0.1–0.2 g/L AP. However, the necessary proliferation of AP at the end of winter is not possible without artificial illumination. Among the various artificial light sources for AP cultivation [42], LEDs are considered to be very promising [34]. Their life cycle is relatively long (up to 50,000 h), and they exhibit low heat generation and high conversion efficiencies. In particular, the selection of certain wavelengths allows for the maximization of light absorption efficiencies and allows one to reach high energy to biomass conversion efficiencies. In comparison to other light sources, LEDs are relatively inexpensive and can operate with a low electrical voltage and current [42,43,44]. Therefore, knowledge about the influence of the spectral composition of the illumination on the growth and biochemistry of AP is crucial for its economic propagation.

In the present study, the illumination of AP with red LED light resulted in the highest biomass yield (+24.50% after 211 h, Figure 3). In contrast, the blue LED light illumination led to a drastic biomass decrease of −56.28% in relation to the cultivation under white LED light. The latter was already observed after 141 h. The relatively low coefficients of variation indicated a good reproducibility between the independent experiments, particularly for those carried out with white LEDs. It is well known that the biomass productivity of AP is related to the photon flux density provided by light, which is captured by the photosynthetic apparatus of the photosynthetic microorganisms [36]. The main light-harvesting structures in AP are phycobilisomes. These are complexes of phycobiliproteins, consisting of protein-linked phycocyanobilins and phycoerythrobilins [45]. Arranged in regular arrays on the thylakoid membrane surface, the phycobilisomes capture light from a relatively wide spectrum [46]. The absorbed light energy is transferred to the reaction centers, which are located in the photosynthetic membranes and converted into chemical energy [47].

Lacking chlorophyll-b, AP uses chlorophyll-a (λa ≈ 430 nm and 680 nm) and accessory phycobiliproteins such as PC (λa ≈ 620 nm) and PE (λa ≈ 570 nm) as light-harvesting protein–pigment complexes [48]. The effect of red LED light is probably due to the fact that this light source emits in the frequency bands of two pigments—chlorophyll-a and PC. Both are abundant in AP, while the other LEDs applied in this study emitted in wavelengths that were relatively far from the peak absorption of any pigment [49]. Thus, it was very likely that the different effects of colored LEDs were responsible for the highest and lowest growth rates of AP under red and blue LED light, respectively.

However, earlier reports about the influence of light color on the quantity and quality of AP biomass are inconsistent. Most studies revealed that red light could induce the strongest growth and blue light the least [34,37,50,51,52,53,54]. In contrast to these results, Chainapong et al. reported a higher growth rate of AP under white light in comparison to blue, red and yellow lights, while Ravelonandro found that the final biomass of AP exposed to green or white light was higher compared to red light [38,55]. In complete contrast to our results, in two studies the highest growth rate was found under blue light; Madhyastha observed the greatest growth in *A. fusiformis* in blue light and the least in red light [36], and Bahman et al. reported that—also for AP—the highest biomasses were observed under blue light [56]. These varying findings might result from differences in the microalgal strains (slight differences are reported even among AP strains), experimental setup and realization of the studies [51]. This may comprise differences in the light sources, cultured AP species (*A. platensis*, *A. maxima* and *A. fusiformis*), composition of the culture medium, aeration rates, pH value, culture temperature and/or illumination periods and intensities.

Previous studies—e.g., with *Porphyra umbilicalis*—revealed that red light could increase cell division and consequently cell density, while blue light could stimulate the accumulation of nitrogen compounds in the form of phycobiliproteins at the expense of cell growth [57]. Our data support these earlier findings, especially for PC in AP. Here, the illumination with blue LED light led to significantly higher PC concentrations compared to white LED light. In contrast, significantly lower PC concentrations were observed after the illumination with green LED light and red LED light (see Table 1B). The pigment phycocyanin is the main photoreceptor of the light-harvesting complex in AP and is responsible for the transfer of the captured light energy to chlorophyl [58,59]. The absorption of the impinging light was mainly in the red frequency range, which effectively promoted photosynthesis. It is well known that cyanobacteria can respond to changes in light color and light quantity by adjusting their relative photosynthetic pigment contents [60,61,62]. This might explain the high PC content in cultures illuminated with blue LED light. Furthermore, the photon flux density was quite low, which, in addition to the illumination with blue LED light [34,36,53], may also have contributed to a high PC concentration [63].

Boussiba and Richmond concluded that blue and green lights may not provide sufficient light energy for the microalgal biomass synthesis [59]. The increases in chlorophyl and phycocyanin contents—but not carotenoids—which occurred in cultures exposed to these light colors were thus considered to be an adaptation to a relatively dark environment and necessary for maintaining biomass generation. However, the results of the present study indicate that these effects also occur when the intensities of the different light colors are equal near the bioreactor surface (here, about 12 µE/(m^2^∙s)).

## 5. Conclusions

In this experimental setup, we used the common production species AP in combination with the widely used Zarrouk medium and investigated the influence of well-defined LED-based light spectra on cyanobacterial growth and phycobiliprotein composition. The generated data can be readily used to adjust AP production for the optimization of yields under routine culture conditions.

In summary, the data of this study revealed that the illumination of AP with blue LED light resulted in the highest phycocyanin concentrations per cell compared to all other LED lights. However, due to the higher AP biomass production under red LED light illumination, the total amount of PC was significantly higher under these conditions compared to all other LED light sources tested. These results suggest that a sequential illumination strategy—with red LED light in the first phase (growth) and blue LED light in the second phase (PC generation)—could be beneficial for optimizing the production of PC in AP. Future studies should clarify whether these results can also be achieved at higher photon flux densities.

## Figures and Tables

**Table 1:** (**A**) Dry biomasses of *Arthrospira platensis* at the end of the cultivation period (*n* = 3 for each experiment). (**B**) Differences of pigment concentrations in relation to the pigment concentration of the respective extracts obtained by white LED light (in percent). Significance levels (*p*) of the statistical analyses of the raw data according to pigment concentrations obtained by white light (*n* = 3 for each experiment).

A.	Dry Weight [g/L]		
LED Color	Experiment 1	Experiment 2	Experiment 3
White	0.38 ± 0.02	0.33 ± 0.03	0.41 ± 0.02
Blue	0.21 ± 0.08		
Green		0.29 ± 0.05	
Red			0.52 ± 0.06
**B.**	**Differences of Pigment Concentrations [%]**		
LED color	Phycocyanin (PC)	Allophycocyanin (APC)	Phycoerythrin (PE)
Δ Blue-White	+15.73 *^p^* ^< 0.001^	−0.675 ^ns^	+14.72 *^p^* ^< 0.001^
Δ Green-White	−8.53 *^p^* ^< 0.001^	+0.92 ^ns^	+3.51 ^ns^
Δ Red-White	−6.97 *^p^* ^< 0.001^	−1.63 ^ns^	−2.44 ^ns^

^ns^ = not significant.

## Data Availability

The data of this study are available from the corresponding author upon reasonable request.
